# Oct4 Contributes to Mesodermal Differentiation by Sustaining the Proliferative Capacity of Early Mesodermal Progenitors

**DOI:** 10.3390/ijms27010054

**Published:** 2025-12-20

**Authors:** Anastasiia V. Lukacheva, Anna S. Zinovyeva, Andrey A. Kuzmin, Mikhail N. Gordeev, Vladislav V. Vasilin, Daria V. Kriger, Nikolay D. Aksenov, Alexey N. Tomilin, Evgeny I. Bakhmet

**Affiliations:** Institute of Cytology, Russian Academy of Sciences, St. Petersburg 194064, Russia; av.lukacheva@yandex.ru (A.V.L.); a.zinoveva@incras.ru (A.S.Z.); a.kuzmin@incras.ru (A.A.K.); sollussekundas@gmail.com (M.N.G.); vlad.vasilin.03@mail.ru (V.V.V.); dkriger@incras.ru (D.V.K.); aksenovn@gmail.com (N.D.A.); a.tomilin@incras.ru (A.N.T.)

**Keywords:** Oct4, pluripotency, embryoid bodies, mesodermal differentiation, cell proliferation, lineage specification

## Abstract

Oct4 is well established as a core regulator of pluripotency, yet emerging evidence points to an additional role in lineage specification during the exit from the pluripotent state. Although Oct4 expression has been observed in early mesodermal progenitors, its precise function in this developmental context remains unclear. To investigate this, we employed embryoid bodies (EBs) as a model of spontaneous differentiation that recapitulates key aspects of early embryonic development in vitro. In accordance with previous studies, reporter assay revealed a distinct temporal pattern characterized by the strong, transient co-expression of *Oct4* and the early mesoderm-specifying marker gene *Brachyury* within a narrow developmental window, consistent with the Oct4 role in early mesodermal progenitors. We further examined the consequences of the Oct4 loss at early stages of this differentiation. Conditional knockout of the *Oct4* gene resulted in a significant reduction in EB size and accumulation of cells in the G0/G1 phase, indicating a critical requirement for Oct4 in maintaining cell proliferation. Despite this defect, cells retained the ability to initiate multilineage differentiation, albeit with reduced expression of *Brachyury* and elevated expression of endodermal markers *FoxA2* and *Sox17.* Interestingly, the formation of beating cardiomyocyte-like structures was also diminished following Oct4 loss and could not be rescued by simply increasing cell numbers. Taken together, these findings highlight an important Oct4 function in mesodermal differentiation, mediated through the maintenance of proliferative capacity of early mesodermal progenitors.

## 1. Introduction

Pluripotent stem cells (PSCs) serve as a cornerstone of developmental biology and regenerative medicine due to their capacity for unlimited self-renewal and their ability to differentiate into all somatic and germline lineages [1,2,3,4]. Understanding the molecular mechanisms that maintain pluripotency while enabling timely lineage commitment is essential for elucidating early embryonic development and for establishing controlled strategies to generate specialized cell types for therapeutic applications [3,5,6]. PSCs originate from the epiblast of the pre- and early post-implantation embryo, where the core transcriptional network is centered around the POU-domain transcription factor Oct4, which is required to sustain the identity, transcriptional plasticity, and developmental potential of pluripotent epiblast (or primitive ectoderm) cells [1,2,7,8,9].

Although Oct4 is classically linked to preservation of the undifferentiated state, accumulating evidence indicates that its function extends to specifying cell fate during PSC differentiation [10,11,12,13]. After implantation, Oct4 expression persists in epiblast cells that begin to exit the pluripotent state and undergo lineage priming to mesendoderm. During this transition, Oct4 regulates the establishment of the embryonic axis, contributes to primitive streak formation, and supports mesendoderm induction through modulation of transcriptional programs associated with Brachyury, Eomes, and Nodal/Smad signaling pathways [10,11,14,15].

Beyond its role in early lineage transitions, Oct4 is a critical regulator of the cell cycle, proliferation, and genome integrity in pluripotent and early differentiating cells. Downregulation or loss of Oct4 leads to G1 accumulation, reduced Cyclin D–CDK4/6 activity, impaired E2F signaling, and overall slowing of cell cycle progression [16,17]. Oct4 directly regulates genes controlling DNA replication, S-phase entry, and mitotic progression, thereby linking transcriptional regulation to proliferative capacity [17,18]. Oct4 also participates in genome surveillance pathways—ATR-dependent phosphorylation of Oct4 prevents DNA-damage-induced cell death during early developmental transitions [18]. Disruption of these mechanisms leads to reduced cell survival and compromised progression through early stages of differentiation [19,20].

Several reports linked Oct4 function with mesodermal and cardiogenic differentiation. Zeineddine et al. demonstrated that Oct4 dosage influences early cardiogenic outcomes through Smad2/4 and promotes the activation of cardiac transcriptional programs during ESC differentiation [21]. Further genomic and functional studies have shown that Oct4 cooperates with WNT/β-catenin signaling and SWI/SNF chromatin-remodeling complexes to regulate mesoderm- and cardiac mesoderm-associated regulatory elements, thereby shaping transcriptional landscapes necessary for early cardiac mesoderm induction [22].

In this study, we performed several functional analyses to further clarify Oct4’s role during the exit from pluripotency and early mesodermal specification. Using an embryoid body (EB) differentiation model, we examined the temporal expression pattern of the *Oct4* and *Brachyury* genes during spontaneous differentiation of mouse ESCs and assessed how conditional *Oct4* gene knockout affects EB size, cell cycle regulation, and differentiation into functional cardiomyocytes during this transition. Our results clearly show that Oct4 is important, though not essential, for mesodermal and cardiogenic differentiation. Its primary function appears to involve the maintenance of proliferation, yet its loss does not prevent PSC differentiation into derivatives of three germ layers, including functional cardiomyocytes.

## 2. Results

### 2.1. Temporal Dynamics of Oct4 and Brachyury Expression During Spontaneous Differentiation

**Figure 1:**
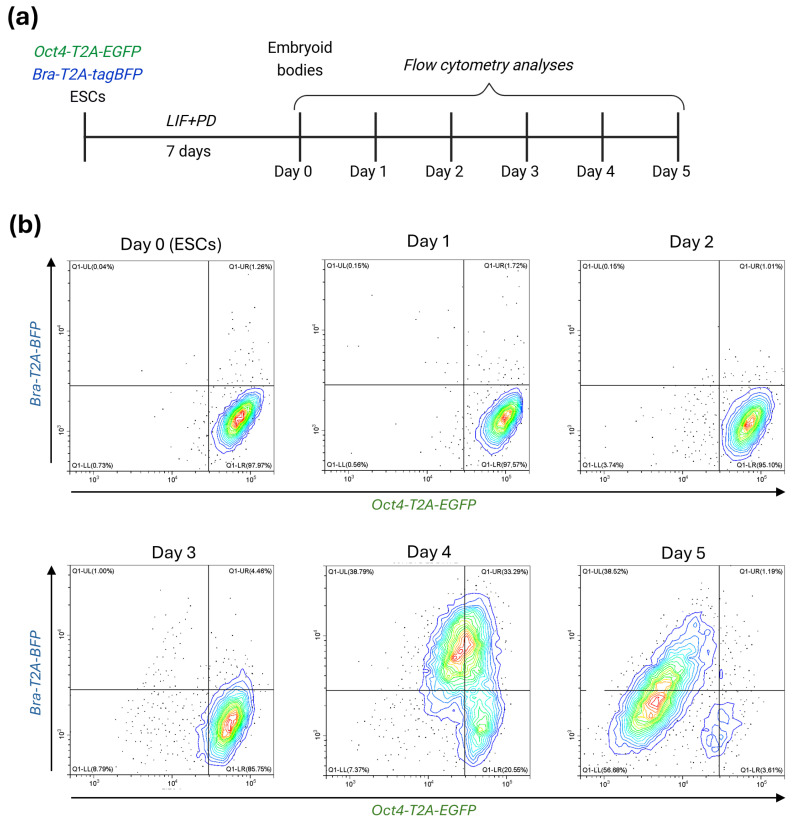
Temporal dynamics of *Oct4* and *Brachyury* (*Bra*) reporter expression during spontaneous differentiation of Ebs: (**a**) Schematic overview of the differentiation workflow: dual-reporter embryonic stem cells (ESCs) carrying *Oct4*-*T2A*-*EGFP*/*Bra*-*T2A*-*tagBFP* reporters were expanded for seven days in the presence of LIF and PD (PD0325901), then aggregated into hanging drops to initiate EB formation and subsequently cultured without LIF and PD for five days. (**b**) Flow cytometry analysis of EGFP and tagBFP expression from Day 0 to Day 5. *Oct4*^+^/*Bra*^−^ cells dominated on Days 0–3 (~85–98%). *Oct4*^+^/*Bra*^+^ population appeared by Day 3 (~4–5%) and peaked on Day 4 (~33%), accompanied by strong induction of *Bra*-only cells (~39%). By Day 5, most cells transitioned to *Oct4*^−^/*Bra*^−^ (~57%) or *Bra*-only (~39%) states, indicating progression beyond the early mesodermal stage. Contour plots reflect density of cells within certain area from blue (low) to red (high).

To trace the *Oct4* expression pattern during the exit from pluripotency and the onset of mesodermal differentiation, we conducted embryoid body (EB)-mediated differentiation of a dual-reporter ESC line harboring *Oct4*-*T2A*-*EGFP* and *Bra*-*T2A*-*tagBFP* constructs knocked into *Oct4* and *Brachyury* genes. The workflow of EB formation and analysis is shown in Figure 1a. Prior to differentiation, cells were exposed to MEK inhibitor PD0325901 to ensure maximal population homogeneity. Equal numbers of cells (600) were then aggregated in hanging drops to initiate EB formation, followed by 5-day culturing without LIF and PD0325901 (Figure 1).

Flow cytometry analysis revealed a clear and orderly progression of EGFP and tagBFP activation during EB differentiation (Figure 1b). On Days 0–2, the population remained largely *Oct4*^+^/*Bra*^−^, due to the transition from naïve to primed pluripotency states, which precedes PSC specification [23]. A measurable shift emerged by Day 3, although *Bra* expression was still minimal (~4–5%). A more pronounced change occurred by Day 4, when a distinct *Oct4*^+^/*Bra*^+^ fraction emerged, marking the onset of primitive streak-like identity. At this timepoint, *Oct4* expression started to decrease, though approximately one-third of all cells were *Oct4*^+^/*Bra*^+^ (~33%), accompanied by strong induction of the *Bra*-only population (~39%), consistent with robust mesodermal specification. By Day 5, both reporters were broadly downregulated, and most cells had transitioned to *Oct4*^−^/*Bra*^−^ or *Bra*-only states, indicating exit from the transient *Oct4*^+^/*Bra*^+^ window and progression beyond early mesoderm.

These data clearly demonstrate that shortly after naïve-to-primed pluripotency transition, *Oct4* is co-expressed with *Brachyury*, indicating a specific function of the former gene at the onset of mesodermal differentiation.

### 2.2. Conditional Loss of Oct4 Impairs EB Growth

**Figure 2:**
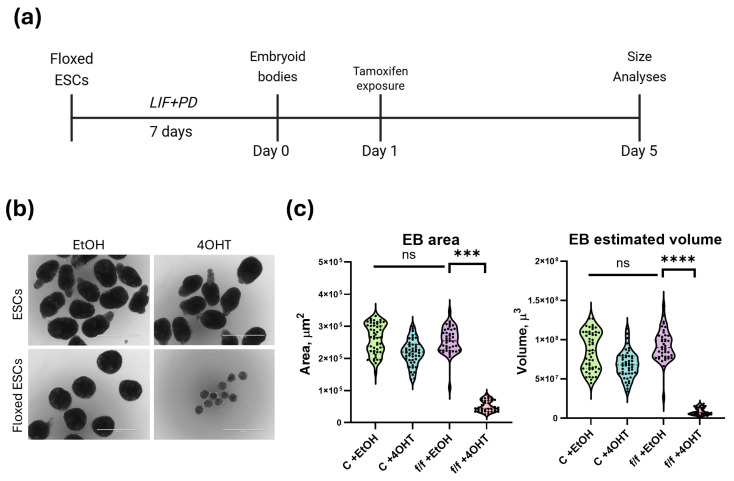
Effects of Oct4 deletion on EB growth: (**a**) Schematic workflow: ESC expansion in LIF + PD0325901, EB formation in hanging drops (Day 0), addition of EtOH or 4OHT on Day 1, followed by assessment of EB growth. (**b**) Representative bright-field images of EBs on Day 5. Control EBs show comparable size under EtOH and 4OHT, while *Oct4*-*floxed* EBs treated with 4OHT are substantially smaller. Scale bars: 1000 μm. (**c**) Left panel: EB area measurements showing a significant reduction in *floxed* + 4OHT EBs compared to *floxed* + EtOH (*** *p* < 0.001) with no difference in the control line (ns). Right panel: EB volume measurements demonstrating a similarly strong decrease in *floxed* + 4OHT EBs (**** *p* < 0.0001) with no significant effect in the control line.

To assess the Oct4 role during early differentiation, we performed conditional knockout of its gene at the onset of differentiation using *Oct4*^*flox*^^/*flox*^; *Rosa*^*26Ert2CreErt2*^ ESCs (floxed, f/f). Oct4 depletion was launched by 4-hydroxytamoxifen (4OHT) exposure, leading to a marked reduction in Oct4 protein level after 24 h and near complete loss by 48 h. To avoid the confounding effect of Oct4 loss during the primed pluripotency phase, 4OHT was added on Day 1 of EB formation. The experimental workflow is shown in Figure 2a. EB morphology and size were assessed on Day 5, corresponding to the peak of proliferative expansion.

Control EBs formed compact spherical aggregates under both EtOH and 4OHT conditions (Figure 2b). Quantitative analysis showed no significant difference in EB area (Figure 2b) and volume (Figure 2c), confirming that 4OHT exposure alone did not affect EB growth. In contrast, *floxed* EBs exhibited a strong dependence on Oct4 expression. EtOH-treated *floxed* EBs were comparable to controls, but 4OHT-treated *floxed* EBs were significantly smaller. Measurements revealed a 5–10-fold reduction in both EB area and volume (*p* < 0.001) (Figure 2c, accompanied by a dramatic decrease in total cell number—from 12–35 × 10^3^ in EtOH-treated samples to 1.5–2.5 × 10^3^ in Oct4-deficient EBs.

These findings indicate that Oct4 is required for maintaining proliferative capacity during early differentiation. The marked reduction in EB size and cell number following Oct4 loss highlights its critical role in supporting the expansion and survival of progenitor populations during the exit from the pluripotency state.

### 2.3. Oct4 Loss Alters Cell Cycle Regulator Expression and Causes G1 Accumulation with Reduced S-Phase Progression

**Figure 3:**
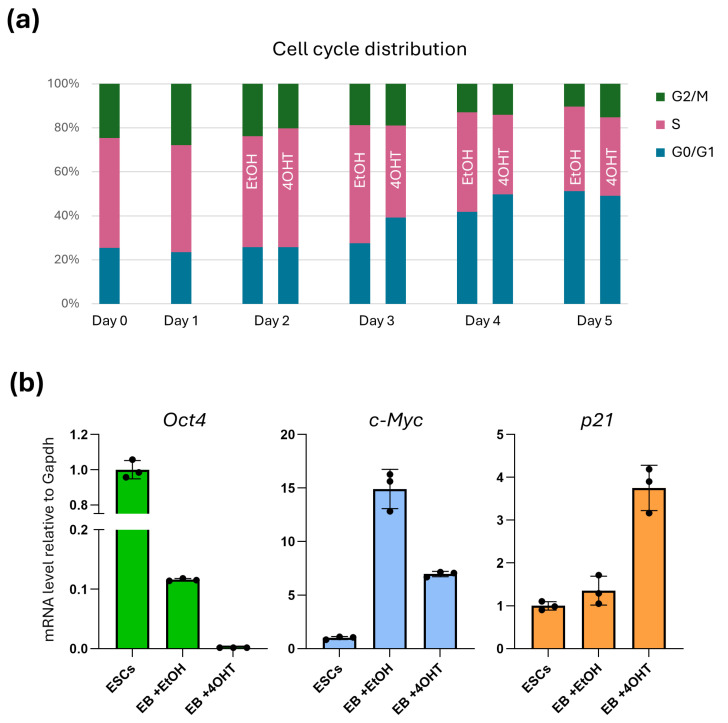
Cell cycle alterations and gene expression changes following Oct4 depletion during early EB differentiation: (**a**) Cell cycle phase distribution (G0/G1, S, and G2/M) in *Oct4*-*floxed* ESCs (Day 0) and EBs (Days 1–5) cultured under EtOH (control) or 4OHT conditions. Oct4 depletion leads to progressive G1 accumulation and reduced S-phase entry beginning on Day 3, coinciding with the expected completion of recombination. (**b**) Relative expression levels of *Oct4A*, *cMyc*, and *p21* mRNA assessed in ESCs (Day 0) and in Day 4 EBs under control (EtOH) or *Oct4* deletion-inducing (4OHT) conditions. The latter conditions promoted near-complete loss of Oct4, impaired *cMyc* induction, and enhanced upregulation of *p21* mRNAs. Gene expression values are presented as ΔCt. Data are shown as mean ± SEM, *n* = 3 technical replicates, presented data reflects average transcript level across 20–100 EBs.

To characterize how Oct4 deletion influences cell cycle dynamics during early embryoid body (EB) differentiation, we first examined DNA-content profiles in *floxed* ESCs on Day 0 and in newly formed EBs on Day 1 (Figure 3a). As no treatment had yet been applied, both time points represent Oct4-intact cells. The cell cycle distributions at D0 and D1 were highly similar, with a predominance of S-phase cells (~49–50%) and a smaller G0/G1 population (~23–25%), reflecting the rapid cycling characteristic of pluripotent cells. These results indicate that the transition from monolayer culture to EB formation does not immediately alter cell cycle progression.

On Day 1, EtOH (control) and 4OHT were added to the EB cultures. Accordingly, no substantial differences between conditions were detected on Day 2 (Figure 3a): EtOH-treated EBs exhibited G0/G1 and S-phase fractions of 25.8% and 50.4%, respectively, whereas 4OHT-treated EBs showed comparable values (25.8% and 53.9%). From Day 3 onward, coinciding with the expected completion of *Oct4* deletion, pronounced differences began to emerge (Figure 3a). Control EBs maintained a proliferative profile typical of early differentiating ESCs (G0/G1 = 27.6%, S = 53.7%), whereas Oct4-deficient EBs exhibited a substantial shift toward G0/G1 accumulation (39.1%) and a corresponding reduction in S-phase entry (41.9%). These effects intensified by Day 4, with G0/G1 sub-population increasing to 49.7% in 4OHT-treated EBs compared to 41.8% in controls, and S-phase decreased to 36.2% versus 45.2%. The altered distribution persisted on Day 5 (4OHT: G0/G1 = 49.1%, S = 35.7%; EtOH: G0/G1 = 38.4%, S = 51.1%), indicating a sustained impairment of DNA synthesis and overall cell cycle progression.

To determine whether the observed changes in cell cycle distribution were associated with transcriptional alterations, we quantified *Oct4*, *cMyc*, and *p21* expression in ESCs (Day 0) and in EBs on Day 4 (Figure 3b). As expected, *Oct4* transcripts were completely eliminated in 4OHT-treated EBs, whereas control Day 4-samples retained low but detectable levels relative to Day 0 (Figure 3b). Loss of Oct4 was accompanied by a pronounced reduction in *cMyc* expression: while *cMyc* was strongly induced in control EBs on Day 4, its levels remained markedly lower in Oct4-deficient samples (Figure 3b). In contrast, *p21* expression was significantly elevated in 4OHT-treated EBs, exceeding levels detected in both Day 0 cells and Day 4 controls (Figure 3b). This reciprocal regulation of *cMyc* and *p21* aligns with the observed deceleration of cell cycle progression and impaired G1/S transition in Oct4-depleted cells.

Altogether, these findings demonstrate that Oct4 is required to sustain the proliferative and transcriptional programs characteristic of early EB differentiation. Loss of Oct4 disrupts the balance of cell cycle regulators, leading to reduced *cMyc* induction, elevated *p21* expression, and a progressive shift toward G1 accumulation with diminished S-phase. These coordinated molecular and cellular changes highlight the essential role of Oct4 in maintaining rapid cell cycle progression during the earliest stages of differentiation.

### 2.4. Oct4 Loss Does Not Block Multilineage Differentiation

**Figure 4:**
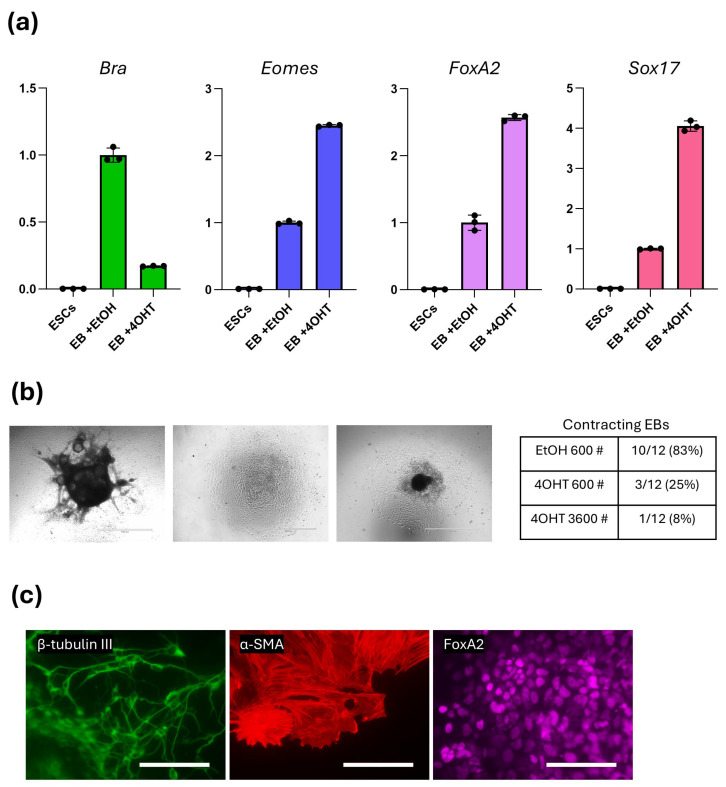
Oct4 loss does not abolish the multilineage differentiation of Ebs: (**a**) qPCR analysis of lineage regulators on Day 4 shows altered quantitative responses upon *Oct4* deletion: reduced *Brachyury* (*Bra*) and increased *Eomes*, *FoxA2*, and *Sox17* mRNAs. (**b**) Left panel: representative bright-field image of EBs outgrowth derived from a control (EtOH) (left), 4OHT-treated *floxed* EBs with normal (600, center) or high (3600, right) number of cells. Scale bars = 1000 µm. Right panel: table represents ratio of contracting EBs versus all EBs (**c**) Immunocytochemistry of EB outgrowths reveals cells positive for β-tubulin III, α-SMA, and FoxA2 proteins. Scale bars: 400 µm.

To evaluate whether Oct4 loss alters the differentiation of EBs, we first examined transcriptional activation of early lineage regulators on Day 4 in control (EtOH) or Oct4-depleted (4OHT) EBs (Figure 4a). In EtOH-treated *floxed* EBs, mesodermal markers *Brachyury* and *Eomes* were strongly upregulated relative to Day 0, consistent with normal primitive streak-like induction. Upon Oct4 deletion, *Brachyury* expression was markedly reduced, whereas *Eomes* level was increased, indicating an imbalance within the early mesendodermal gene network. Interestingly, endoderm-associated genes showed similar expression upregulation: *FoxA2* and *Sox17* were both elevated in 4OHT-treated EBs (Figure 4a). These data indicate that Oct4 is not essential for initiating lineage programs but is required to maintain the proper quantitative balance of germ-layer transcriptional outputs.

To assess downstream differentiation potential, Day 5 EBs were transferred to gelatin-coated plates and cultured for an additional three days. To compensate for the reduced proliferation and smaller size of 4OHT-treated EBs, an additional experimental condition with six-fold more cells (3600), also treated with 4OHT, was included. Outgrowths derived from Oct4-deficient EBs were small and displayed flattened morphology (Figure 4b). Although higher cell numbers produced larger EBs, they did not reach the size of control EBs. Surprisingly, examination for beating cardiomyocyte-structures revealed that contractions still occurred in Oct4-depleted EBs, but with substantially lower frequency and smaller contracting area (Figure 4b, Supplementary Videos). However, increasing cell numbers failed to rescue this phenotype, resulting in even lower beating structures (Figure 4b, Supplementary Videos).

Immunocytochemical analysis revealed the presence of differentiated cells expressing βIII-tubulin, α-smooth muscle actin (α-SMA), and FoxA2 (Figure 4c). These markers represent derivatives of ectodermal, mesodermal, and endodermal lineages, demonstrating that multilineage differentiation capacity was preserved despite the pronounced growth defects caused by Oct4 deletion.

Overall, Oct4 loss does not prevent pluripotency exit or germ layer initiation, but it disrupts the balanced activation of lineage regulators and compromises functional maturation, likely reflecting insufficient proliferative expansion of early mesodermal progenitor pools.

## 3. Discussion

Our study reveals that Oct4 plays a fundamental role in maintaining the proliferative activity of differentiating cells at the early stages of development. Using an EB formation model, we showed that Oct4 expression persists during the transition from pluripotent state to mesodermal lineage commitment, and that *Oct4* deletion results in a pronounced reduction in EB size and total cell number, accumulation of cells in the G1 phase, and decreased S-phase entry. At the same time, the ability of cells to generate derivatives of different germ layers remained largely intact. These findings indicate that Oct4 acts not as a direct determinant of lineage identity, but as a key regulator sustaining proliferation during the early differentiation process.

The transient *Oct4*^+^/*Brachyury*^+^ population identified in our experiments corresponds to the developmental stage in which pluripotent epiblast cells enter the primitive streak and give rise to mesodermal precursors. This pattern is consistent with in vivo observations showing that *Oct4* expression persists in the primitive streak and nascent mesoderm, where it is required around E7.5 to sustain progenitor proliferation and ensure proper morphogenesis [10,11]. Similar co-expression of *Oct4* and *Brachyury* has been observed in EB differentiation models that combine live imaging with molecular profiling, further confirming that Oct4 activity transiently overlaps with Brachyury induction during early mesodermal commitment [24,25]. Such a state represents a short-lived phase of developmental plasticity in which progenitor cells maintain Oct4-dependent proliferative programs while activating early lineage genes.

Loss of Oct4 leads to growth retardation and accumulation of cells in G1, reflecting a defect in cell cycle progression similar to that observed in Oct4-deficient embryos and ESCs [11,16]. In human PSCs, Oct4 phosphorylation by ATR and other kinases stabilizes the protein under replication stress and couples checkpoint control to proliferation [18]. Similar mechanisms may operate in mesodermal progenitors as well, where Oct4 ensures the balance between lineage transition and continued growth.

Despite the marked decrease in growth, Oct4-deficient cells retained the capacity to initiate mesodermal, endodermal, and ectodermal differentiation, as evidenced by the expression of α-smooth muscle actin, FoxA2, and β-tubulin III. On the other hand, it is obvious that the balance in lineage specification was shifted as the RNA level of mesodermal inducer *Brachyury* was severely decreased, while *Eomes* and the endodermal markers *FoxA2* and *Sox17* were elevated. This pattern is consistent with previous reports pointing to the Oct4 role in mesodermal specification [12,22,26,27]. It is worth noting that according to previous data [21], cardiac differentiation was severely affected, but not completely cancelled under total Oct4 loss. Consequently, Oct4 can be viewed as a permissive rather than instructive regulator—it enables expansion and survival of early progenitors but cannot act as a lineage inducer in the manner of Brachyury. Thus, it seems that Oct4 does not undergo a total functional shift at the onset of gastrulation; instead, it appears to preserve its role in maintaining the proliferation of mesodermal progenitors. Once the remaining components of the pluripotency network are shut down, Oct4 correspondingly loses its function as a gatekeeper of the undifferentiated state.

Although our study provides new insight into the role of Oct4 during early differentiation, several limitations should be acknowledged. First, most of our analyses were performed using the EB model, which, despite its value in recapitulating early developmental events, cannot fully reproduce the spatial organization and signaling complexity of the embryo. Second, although we demonstrate clear changes in proliferation upon Oct4 loss, the underlying transcriptional and signaling mechanisms were not directly examined. Future studies incorporating transcriptomic or chromatin profiling approaches will be necessary to define the molecular networks through which Oct4 controls these processes.

## 4. Materials and Methods

### 4.1. Plasmid Construction

The tagBFP-coding sequence was amplified from a plasmid pTagBFP-N vector (Evrogen, Moscow, Russia) and cloned into the *pOct4*-*TA2*-*EGFP* vector, using SgrAI/MluI restriction sites, to replace the EGFP sequence, resulting in the *Oct4*-*TA2*-*tagBFP* vector. The left and right homology arms (727 bp and 911 bp, respectively), corresponding to regions adjacent to the *Bra* (*T*) gene stop codon, were amplified from mouse genomic DNA using Phusion DNA polymerase (ThermoFisher, Waltham, MA, USA). The left homology arm was digested with AvrII/SbfI. This fragment was then ligated into the AvrII/NsiI-digested *Oct4*-*TA2*-*tagBFP* backbone. Next, the right homology arm was digested with MluI/SalI and cloned at the corresponding sites of the vector to complete the *Bra*-*TA2*-*tagBFP* donor plasmid.

The guide-RNA sequences for the CRISPR-mediated DNA double-strand breaks were selected using the Benchling platform (https://www.benchling.com/) within the sequence around the stop codon of the mouse *Bra* gene. The selected guide RNA had an optimal On-/Off-target score, according to the method proposed by Hsu et al. [28]. The chosen guide-RNA was purchased from Evrogen (Russia), annealed, and ligated into the LentiCRISPRv2 vector (Addgene, Watertown, MA, USA). All the final constructs were verified by Sanger sequencing. Table 1 lists all the oligonucleotides used for the above manipulations.

### 4.2. ESC Lines

E14 Tg2a murine ESCs (Bay Genomics) were used as wild-type ESCs. *Oct4*-*T2A*-*EGFP* mouse embryonic stem cells (ESCs), derived from the E14 ESCs, were described elsewhere (https://www.biorxiv.org/content/10.1101/2024.09.07.611681v2, accessed on 11 December 2025). To generate double reporter cells, *Oct4*-*T2A*-*EGFP* ESCs were further transfected with *Bra*-*TA2*-*tagBFP* donor vector and *LentiCRISPRv2* plasmid harboring gRNA (500 ng with a 1:1 molar ratio) using FuGENE HD transfection reagent (Promega, Madison, WI, USA) in OptiMEM medium. The next day, cells were transferred onto a 10 cm gelatinized dish. One day later, 2 μg/mL puromycin (Merck, Darmstadt, Germany) was added to the culture medium for two additional days. The cells were cultured for an additional 10 days without the selective antibiotics. Then, single clones were picked, expanded, and tested for transgene insertion by PCR using the gtM_T_F2.1 and gtM_tagBFP_R2.1 primers with the LR HS-PCR kit (Biolabmix, Novosibirsk, Russia).

The *Oct4*^*flox*/*flox*^; *Rosa*^*26Ert2CreErt2*^ “floxed” ESC line was derived from the *Oct4*^*flox*/*flox*^ ESCs [29] by inserting the Ert2-Cre-Ert2 fragment into the *Rosa26* locus, using previously designed and described DNA constructs [30].

### 4.3. Cell Culture

Unless otherwise specified, all reagents for mouse ESC culturing were purchased from Gibco (ThermoFisher Scientific, Waltham, MA, USA). ESCs were routinely cultured on adhesive plastic dishes (Eppendorf or TPP), precoated with 0.1% gelatin (Merck) under standard conditions (5% CO_2_, 37 °C). The mES medium consisted of Knockout DMEM supplemented with 15% fetal bovine serum (HyClone), 1× penicillin/streptomycin, 2 mM L-Glutamine, 1× non-essential amino acids, 50 μM β-mercaptoethanol, and 500 U/mL bacterially expressed human LIF produced in-house. Passaging was performed using 0.05% Trypsin-EDTA.

Prior to the experiments, all three ESC lines were pre-cultured for 7 days in medium containing LIF and 1 μM PD0325901 (Axon, Scottsdale, AZ, USA) to stabilize the pluripotent state and to get rid of spontaneous differentiation. Oct4 knockout was induced by adding 0.8 μg/mL 4-hydroxytamoxifen (4OHT, Merck) to the medium.

### 4.4. EB Formation and Differentiation

Single-cell suspensions of ESCs were prepared by 0.05% Trypsin-EDTA dissociation and seeded as 600 cells per 35 μL hanging drop to initiate EB formation (Day 0). The culture medium for EB generation was the same as that used for ESC maintenance, except that LIF and PD0325901 were omitted. After 24 h (Day 1), ethanol (EtOH) or 4-hydroxytamoxifen (4-OHT) dissolved in ethanol was added (final volume 40 μL) to EBs formed from the control and *floxed* ESC lines to induce Oct4 knockout. This timepoint was chosen to keep Oct4 expression intact at the stage of primed pluripotency, as the 4OHT effect is visible only after 24 h after addition to ESCs ((https://www.biorxiv.org/content/10.1101/2024.09.07.611681v2, accessed on 11 December 2025)). No treatment was applied to the *Oct4*-*T2A*-*EGFP*/*Bra*-*T2A*-*tagBFP* EBs. When indicated, at Day 5, EBs were plated onto 48-well gelatin-coated plates in DMEM/F12 GlutaMAX + 10% FBS + penicillin/streptomycin. EBs were cultured for an additional 3 days to monitor spontaneous contractile activity. Beating EBs were recorded by screen capture using EVOS fl Auto microscope (ThermoFisher Scientific, Waltham, MA, USA), then fixed for immunocytochemical analysis.

### 4.5. Flow Cytometry

Flow cytometric analyses were performed to assess reporter gene expression dynamics in *Oct4*-*T2A*-*EGFP*/*Bra*-*T2A*-*tagBFP* ESCs during spontaneous differentiation of ESCs into EBs and to analyze cell cycle distribution in *floxed* ESCs treated with either EtOH or 4OHT during the same differentiation process.

Flow cytometry was conducted with a CytoFLEX benchtop flow cytometer (Beckman Coulter, Brea, CA, USA) equipped with 405 nm and 488 nm lasers. The 405 nm laser was used for DAPI and BFP detection, and the 488 nm laser for EGFP detection (FITC channel). For reporter expression analysis, fluorescence intensities of EGFP and BFP were measured at multiple time points (Day 0–Day 5) during differentiation. Debris and doublets were excluded by forward/side scatter gating. For cell cycle analysis, 15 μL of a mixture containing DAPI (100 μg/mL) and 1% Triton X-100 was added to 150 μL of the cell suspension before flow cytometry acquisition. Data from all assays were processed in CytExpert (version 2.4, Beckman Coulter).

### 4.6. Immunocytochemistry and Imaging

Cells were fixed with 4% paraformaldehyde for 10 min directly on culture plates, washed with PBS plus 0.1% Tween (PBST), permeabilized by 0.1% Triton X-100 for 15 min, and blocked with 3% BSA-PBST for 30 min. Incubation with the primary antibodies, diluted in 3% BSA-PBST, was performed for 2 hr at room temperature or overnight at +4 °C. Cells were washed 5–6 times with PBST and incubated with secondary fluorescent antibodies diluted in 3% BSA-PBST for 1 hr at room temperature. Afterwards, cells were washed 3 times with PBST, incubated with DAPI for 2 min, washed, and stored in PBS with sodium azide. All primary antibodies used in this study are listed in Table 2. Secondary fluorescent antibodies were purchased from Jackson ImmunoResearch (West Grove, PA, USA). Fluorescent microscopy was performed using an EVOS fl Auto (ThermoFisher Scientific, Waltham, MA, USA) microscope equipped with DAPI, GFP, RFP, and CY5 filter cubes.

### 4.7. Gene Expression Analysis by Real-Time Quantitative PCR

Total RNA was isolated from cells using the guanidinium thiocyanate–phenol–chloroform extraction method with the RNA-extract reagent (Evrogen, Russia) according to the manufacturer’s instructions. Complementary DNA (cDNA) was synthesized from 2 µg of total RNA using random hexamer primers and MMLV reverse transcriptase (Evrogen, Russia).

Real-time PCR was performed with 5× qPCRmix-HS SYBR (Evrogen, Russia) on an ABI 7500 Real-Time PCR System (Thermo Fisher Scientific, Waltham, MA, USA). Gene expression levels were normalized to *Gapdh* expression. Primer sequences are listed in Table 3.

### 4.8. Statistical Analysis

All datasets were statistically analyzed using the GraphPad Prism v8.4.2 package (GraphPad Software, San Diego, CA, USA). Data distribution normality was tested using the Shapiro–Wilk and Kolmogorov–Smirnov tests. Depending on normality, group differences were evaluated using either ordinary one-way ANOVA with Tukey’s multiple comparisons test or the Kruskal–Wallis test for non-parametric data. A *p*-value < 0.05 was considered statistically significant. Quantitative analysis of EB morphology was performed using Fiji (version 1.54, ImageJ, NIH, Bethesda, MD, USA). The area and volume of individual EBs were measured manually from bright-field micrographs acquired on Day 5 of differentiation. Each EB was outlined using the freehand selection tool, and its area was recorded automatically. EB volume was estimated assuming spherical geometry based on the measured two-dimensional area. For each experimental condition, at least 20 EBs were analyzed per biological replicate.

## Figures and Tables

**Table 1:** List of oligonucleotides used for construction and validation of the T-TA2-tagBFP reporter vector and CRISPR/Cas9 components.

Oligonucleotide Name	Sequence (5′→3′)
cV_BFP_F1	TATATcaccggtgatgagcgagctgattaaggagaac
cV_BFP_R1	TATACGCGTattaagcttgtgccccagtttg
cM_LA-T_F2	tatcctaggAGTCATGGGGCAAGGTCAAG
cM_LA-T_R1	tatctgcagCATAGATGGGGGTGACACAG
cM_RA-T_F1	aaaacgcgttagGGCTCAAAGTGGCAGGCTCT
cM_RA-T_R1	aaagtcgacGGCAGACAGATACCTATGGCAA
T_Guide#2_F	caccgCTAGAAGATCCAGTTGACAC
T_Guide#2_R	aaacGTGTCAACTGGATCTTCTAGc
gtM_T_F2.1	GTCCGAATGCCTTTGTAGATGC
gtM_tagBFP_R2.1	gcatgttctccttaatcagctcg

**Table 2:** List of primary antibodies used in this study.

Target	Cat. No.	Manufacturer
*Oct4* (C-10)	sc-5279	Santa Cruz Biotechnology (Dallas, TX, USA)
α-SMA	A2547	Sigma-Aldrich (St. Louis, MO, USA)
βIII-Tubulin	MMS-435P	Covance (Princeton, NJ, USA)
FoxA2	Sc-374375	Santa Cruz Biotechnology

**Table 3:** Primer sequences.

Gene	Primer Sequence (Forward)	Primer Sequence (Revere)
*Oct4A*	GGCTTCAGACTTCGCCTTCT	TGGAAGCTTAGCCAGGTTCG
*Brachyury*	GGCTGGGAGCTCAGTTCTTT	AGCAGCCCCTTCATACATCG
*Eomes*	AAGCTCAAGAAAGGAAACATGC	ACCGGCACCAAACTGAGA
*FoxA2*	CATGAACTCGATGAGCCCCA	TGTAGCTGCGTCGGTATGTC
*Sox17*	CACAACGCAGAGCTAAGCAA	CGCTTCTCTGCCAAGGTC
*c-Myc*	CTGGAGATGATGACCGAGTTAC	GAGAAACCGCTCCACATACA
*p21* *Gapdh*	TCGCTGTCTTGCACTCTGGTGT AGGTCGGTGTGAACGGATTTG	CCAATCTGCGCTTGGAGTGATAG TGTAGACCATGTAGTTGAGGTCA

## Data Availability

The original contributions presented in this study are included in the article/Supplementary Material. Further inquiries can be directed to the corresponding author.

## Supplementary Materials

The following supporting information can be downloaded at: https://www.mdpi.com/article/10.3390/ijms27010054/s1.
